# The Gene Encoding NAD-Dependent Epimerase/Dehydratase, *wcaG*, Affects Cell Surface Properties, Virulence, and Extracellular Enzyme Production in the Soft Rot Phytopathogen, *Pectobacterium* *carotovorum*

**DOI:** 10.3390/microorganisms7060172

**Published:** 2019-06-13

**Authors:** Rabiul Islam, Shyretha Brown, Ali Taheri, C. Korsi Dumenyo

**Affiliations:** Department of Agricultural and Environmental Sciences, Tennessee State University, 3500 John A. Merritt Blvd; Box 9543, Nashville, TN 37209, USA; gb5993@wayne.edu (R.I.); shydslow@aol.com (S.B.); ataheri1@tnstate.edu (A.T.)

**Keywords:** *Pectobacterium*, exopolysaccharide, cell surface properties, *O*-specific antigen, biofilm, colanic acid, soft rot

## Abstract

*Pectobacterium carotovorum* is a gram-negative bacterium that, together with other soft rot Enterobacteriaceae causes soft rot disease in vegetables, fruits, and ornamental plants through the action of exoproteins including plant cell wall-degrading enzymes (PCWDEs). Although pathogenicity in these bacteria is complex, virulence levels are proportional to the levels of plant cell wall-degrading exoenzymes (PCWDEs) secreted. Two low enzyme-producing transposon Tn5 mutants were isolated, and compared to their parent KD100, the mutants were less virulent on celery petioles and carrot disks. The inactivated gene responsible for the reduced virulence phenotype in both mutants was identified as *wcaG*. The gene, *wcaG* (previously denoted *fcl*) encodes NAD-dependent epimerase/dehydratase, a homologue of GDP-fucose synthetase of *Escherichia coli*. In *Escherichia coli*, GDP-fucose synthetase is involved in the biosynthesis of the exopolysaccharide, colanic acid (CA). The *wcaG* mutants of *P. carotovorum* formed an enhanced level of biofilm in comparison to their parent. In the hydrophobicity test the mutants showed more hydrophobicity than the parent in hexane and hexadecane as solvents. Complementation of the mutants with extrachromosomal copies of the wild type gene restored these functions to parental levels. These data indicate that NAD-dependent epimerase/dehydratase plays a vital rule in cell surface properties, exoenzyme production, and virulence in *P. carotovorum*.

## 1. Introduction

Soft-rot disease is caused by *Pectobacterium* and *Dickeya* genera of bacteria which are usually referred to as soft rot Enterobacteriaceae (SRE). Species in the two genera were previously classified under the genus, *Erwinia* before subsequent revisions assigned them to *Pectobacterium* and *Dickeya* genera based on their host range, biochemical and molecular traits [1,2]. Soft rot Enterobacterieaceae secrete large amounts of exoproteins including plant cell wall-degrading enzymes (PCWDEs) such as pectate lyases (Pel), polygalacturonases (Peh), proteases (Prt), and cellulases (Cel) [3,4]. The exoproteins produced by SREs are secreted through three secretion systems: pectinolytic and cellulolytic enzymes such as pectate lyase and cellulase are secreted through the type II secretion system (T2SS) [5], proteases are secreted through the type I system [6], and harpins and other effectors through the type III system [7]. During infection, these enzymes degrade the plant cell wall structures resulting in the maceration and soft rot of the host tissue. The level of these enzymes produced is positively correlated with bacterial virulence [8].

The production of plant cell wall-degrading enzymes (PCWDEs) and therefore virulence of *Pectobacterium* is controlled by a combination of bacterial regulatory genes (such as *rsmA*, *rsmB*, *rsmK*, *gacA*, *expR1*, *expR2*, *hexA*, *ahl*, *kdgR*, *hrpL*) and host chemical signal molecules generally referred to as inducers [4]. For example, the RNA-binding RsmA protein and noncoding RsmB RNA control the production of extracellular enzymes (PCWDEs), antibiotics, pigments, polysaccharides, the synthesis of flagella, and the levels of the quorum sensing signal, acyl homoserine lactone in various *Pectobacterium* species [9,10,11,12,13,14].

Colanic acid (CA) is an extracellular polysaccharide (EPS) found in many species within Enterobacteriaceae [15]. Unlike lipopolysaccharide (LPS) and capsular polysaccharide (CPS), both of which are closely associated with bacterial surface, CA is loosely associated and forms saccharide mesh. In *Escherichia coli*, CA is a polymer of repeating subunits of D-glucose, L-fucose, D-galactose, and D-glucouronic acid and two of these sugars are covalently linked with O-acetyl and pyruvate. The assembly of mature CA follows identical processes as O antigen of lipopolysaccharide [16,17]. In some cases, especially in highly mucoid *Escherichia coli* strains, CA repeats connect to the core region of LPS forming modified LPS (MLPS) [18]. In *Escherichia coli*, the product of *wcaG* gene which is part of the *wca* operon is a dual function enzyme specifying GDP fucose synthetase, a dual function including 3,5-epimerase-4-reductase involved in GDP fucose biosynthesis. GDP fucose synthesis proceeds through a two-step enzymatic reaction from GDP mannose [19].

A homologue of *wcaG* gene is also found in *Pectobacterium carotovorum* that encodes GDP fucose synthetase-like protein and has been annotated as NAD-dependent epimerase/dehydratase. Recently, the exopolysaccharide, colanic acid (CA) and lipopolysaccharide (LPS) have been reported to be associated with virulence in *Pectobacterium* as the purified molecules induced pathogenicity-related physiological responses such as cell death and generation of reactive oxygen species in host cells [20]. Here, we demonstrate that mutation in *wcaG* gene in *Pectobacterium carotovorum* strain Ecc71 reduces PCWDEs production and virulence remarkably and affects cell surface properties including biofilm formation and cell surface hydrophobicity. To the best of our knowledge, this report is the first genetic evidence linking colanic acid biosynthesis to the production of PCWDEs and virulence in *Pectobacterium*.

## 2. Materials and Methods 

### 2.1. Bacterial Strains, Media and Growth Conditions

The bacterial strains used in this study are listed in Table 1. *Pectobacterium carotovorum* strains were grown in minimal salts plus sucrose medium (MM) with or without host extracts at 28 °C. *Escherichia coli* was grown in Luria broth (LB) medium at 37 °C. Where required, MM media were supplemented with 30% (*v*/*v*) celery extract (CE) or 0.2% polygalacturonic acid (PGA). Antibiotic drugs were used at the following concentrations in (µg/mL); kanamycin (Km), 50; nalidixic (Nal), 50, tetracycline (Tc), 10. When needed, media were solidified with 1.5% (*w*/*v*) agar before autoclaving.

### 2.2. Mutant Isolation by Transposon Mutagenesis

The procedure for the isolation of KD250 and KD251 has been described [28]. The two mutants, KD250 and KD251 were isolated for having reduced extracellular protease activity in vitro on Nutrient-gelatin agar.

### 2.3. DNA and Other Molecular Techniques

The construction of the genomic library of *P. carotovroum* Ecc71 has been described (Murata 1994). Three pairs of primers for *wcaG* (Table S1) were used to screen the genomic library of Ecc71. The *wcaG^+^* cosmid clone, designated pCKD252 was tri-parentally mated into *wcaG* mutants, KD250 and KD251 to complement the mutations. Transconjugants were selected on double antibiotic (Km and Tc) selection medium.

### 2.4. wcaG Cloning and Sequencing

Oligonucleotide primers were obtained from MWG Operon Biotechnologies (Madison, AL, USA). The primer Tn-LacZ P6 (Table S1) was used to sequence across the transposon junction into the flanking genomic sequence using genomic DNA as template, and the generated DNA sequences were used to search against genomic databases of *Pectobacterium* and *Dickeya* spp. The truncated gene in KD250 and KD251 was designated *wcaG_Ecc71_* in keeping with the nomenclature used in *Escherichia coli.*

### 2.5. Growth Curve of Pectobacterium Strains

Growth curve was plotted according to Zwietering et al. [29]. Briefly bacteria were inoculated into MM or MM + CE and incubated on a shaker at 28 °C. Bacterial growth was measured hourly by culture turbid using a Klett colorimeter. The readings were recorded until the bacteria reached the stationary phase.

### 2.6. Biofilm Assays

Biofilm formation was measured according to the method of Bakke et al. [30]. Bacterial cultures were grown to A_600_ of 0.1 in MM with the appropriate antibiotic drug. One hundred and fifty microlitres of culture was dispensed into each well in a 96-well plate. The plate was sealed with parafilm and incubated in a still incubator without shaking at 28 °C for 20 h. The plantonic cells were removed by rinsing with tap water slowly. Two hundred microlitres of 0.1% crystal violet was added into each well for 15 min to stain the bacteria attached to the wells. The crystal violet was removed, and the wells were rinsed with tap water. Two hundred microlitres of 95% ethanol was added to each well to dissolve the crystal violet. Absorbance at 590 nm was measured using a Synergy H1 hybrid Reader (BioTek, Winooski, VT, USA).

### 2.7. Pathogenicity Test and Bacterial Population Count in Host

A pathogenicity test was performed on celery petioles according to the method of Kersey et al [28]. Tissue maceration of mutants was compared with that of the parent by inoculating celery petioles or carrot disks with 10 µL of bacterial suspension standardized to OD_600_ = 0.2 (approx. 7.6 × 10^8^ CFU). The petioles and discs were incubated in a moisture chamber at 28 °C for 48 h. Macerated tissue from carrot disks was obtained by calculating the difference between the weight before and after washing/wiping the rotten tissue. For bacterial population count, OD_600_ = 0.1 (approx. 3.8 × 10^8^ CFU) of bacterial suspension was inoculated in celery petioles and incubated for 0 to 3 days in a moist chamber. The infected area of the celery petioles was separated with a knife carefully and ground with a mortar and pestle. Ground petioles were serially diluted in 0.5× PBS and plated on LB media.

### 2.8. Quantitative Exoenzyme Assay

Quantitative enzyme assays were performed for Pel and Prt as previously described [28]. Quantitative Pel activity was spectrophotometrically (Synergy H1, Biotek) determined for pectate degradation using polygalacturonic acid as substrate. Assays for Prt activity were conducted using azocasein (2%) as substrate. The enzymatic activities were corrected for bacterial growth measured by optical density at 600 nm.

### 2.9. Hydrophobicity Test

A hydrophobicity test was performed as described by Rosenberg [31] with some modifications. Bacterial strains were grown in liquid MM and diluted to A_600_ = 0.5 using 0.5× Phosphate-buffered saline (PBS). An equal volume (5 mL) of bacterial suspensions and nonpolar solvents (hexane and hexadecane) was mixed in a glass tube and vortexed for 2 min. Glass tubes were allowed to stand for 10 min for phase separation. The optical density of the aqueous phase was measured at 600 nm.

### 2.10. Multiple Sequence Alignment and Phylogenetic Tree Analysis

Amino acid sequences of WcaG of all species were obtained from GenBank (www.ncbi.nlm.nih.gov). Multiple sequence alignment was then performed using Clustal Omega, an alignment platform from European Bioinformatics Institute. A phylogenetic tree was constructed based on the DNA sequences of *wcaG* gene of all species using the Maximum likelihood method in Molecular Evolutionary Genetics Analysis (MEGA) [32].

## 3. Results

### 3.1. Isolation and Characterization of wcaG Mutant

**Figure 1:**
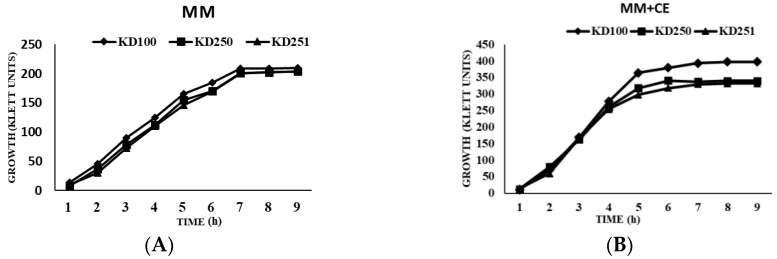
Growth curves of *P. carotovorum* strain KD100 and its *wcaG* mutants KD250, KD251. Bacterial cultures were grown in MM (**A**) and MM + CE (**B**) media. Growth was measured with the Klett colorimeter in Klett units.

**Figure 2:**
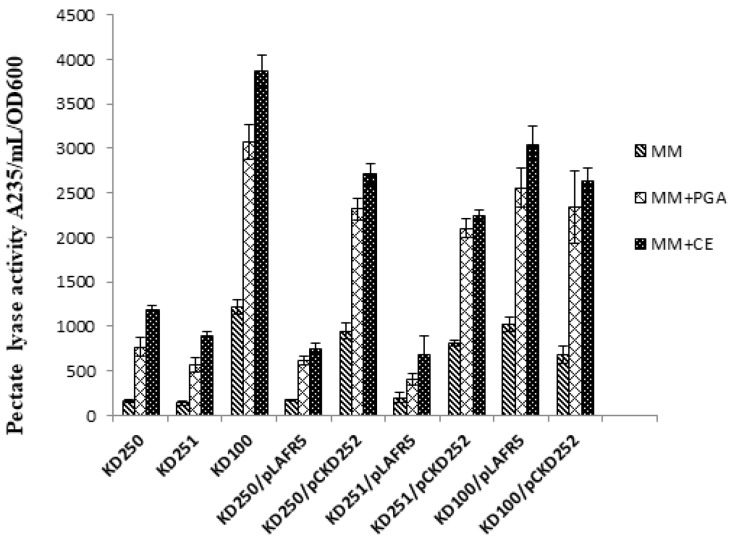
Quantitative assays for enzymatic activities of extracellular pectate lyase from *wcaG^+^* and *wcaG^−^ P. carotovorum* strains. Cultures were grown in liquid MM, MM + CE and MM + PGA at 28 °C for 16 h. Pel activities were determined from cultural supernatant. The cosmid, pCKD252 (carrying wild type *wcaG* clone in pLAFR5) was used to complement the mutants KD250, KD251. Values are the mean (from four replicates) ± standard deviation.

**Figure 3:**
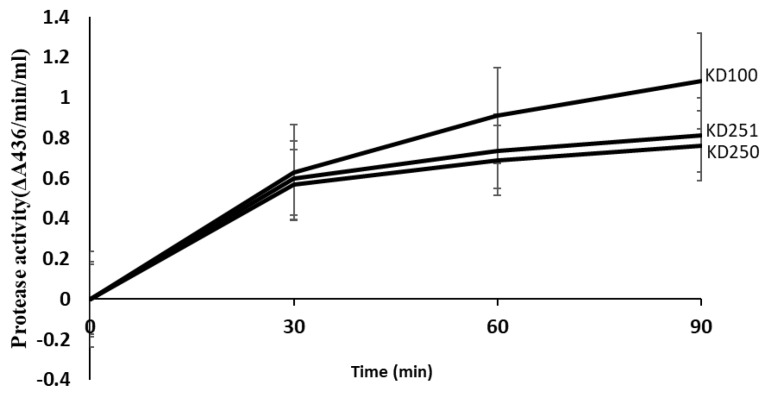
Quantitative assays for enzymatic activities of extracellular protease from *P. carotvorum* strain KD100 and its *wcaG^−^* mutants KD250 and KD251. The parental strain, KD100 and *wcaG* mutants KD250, KD251 were cultured (three replicates were taken from culture tube of each strain) in MM supplemented with celery extract. Supernatants of these cultures were used to measure protease activities using azocasein as a substrate. Values are the mean (from three replicates) ± standard deviation.

Random mutagenesis was performed on *P. carotovorum* strain KD100 by using mini- Tn5 *lacZ*1 to isolate the mutant with altered levels in extracellular protease (Prt) production on nutrient gelatin (NG) medium [28]. Several mutants were selected of which the mutants, designated KD250 and KD251 showed low extracellular protease activity (less than 0.5-fold) compared to the parental level. The growth of the mutants KD250, KD251 was similar to that of the parent KD100 (Figure 1). The mutants even had a slightly shorter log phase in host extract (CE)-supplemented medium. We assayed for Pel (Figure 2) and Prt (Figure 3) activities quantitatively from the culture supernatants when the mutant KD250, KD251 and parent KD100 were grown in MM, MM with celery extract (MM + CE) and MM with polygalacturonic acid (MM + PGA). As expected, in comparison to MM medium, the host extract-supplemented media induced the bacteria to produce more Pel and Prt in both the mutants and parent. This also suggests that the mutants are still responsive to induction by the signal from the host extract. However, the level of exoenzymes in the mutants was still low in comparison to parent KD100.

### 3.2. Identification and Characterization of wcaG_Ecc71_

**Figure 4:**
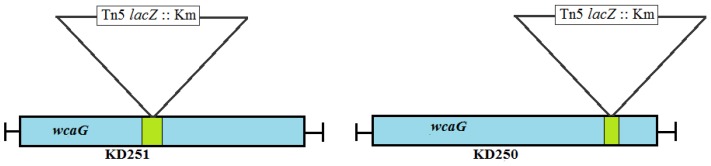
Schematic of transposon insertion in the mutants. Insertion point of transposon *lacZ*1 Km (2.36kb) in *wcaG* of both KD250 and KD251.

**Figure 5:**
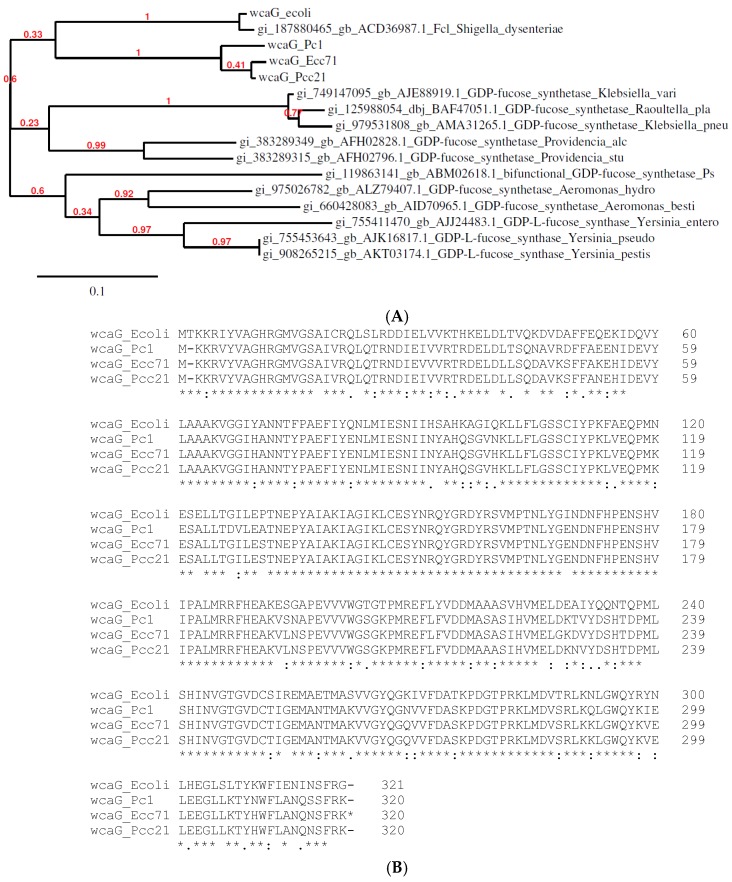
(**A**) Phylogenetic tree of predicted *P. carotovorum* Ecc71 WcaG with other Enterobacteriaceae family members. The phylogenetic tree reveals that the WcaG protein of *Escherichia coli* is evolutionarily close to those from *Pectobacterium* species. (**B**) Multiple sequence alignment of WcaG protein sequences from *Escherichia coli* and *Pectobacterium* strains. The alignment was made with deduced amino acid sequences of WcaG of *P. carotovorum* Ecc71 and its homologs from strains PC1, PCC21 and *Escherichia coli* K12. The alignment reveals almost similar or identical deduced amino acid sequences.

We sequenced the genomic DNA of the mutants KD250, KD251 across the transposon junction to determine the transposon insertion region and what gene might have been truncated. Interestingly, the transposon inserted in the different region of same gene (Figure 4) in both mutants KD250 and KD251. Based on the sequence in this region, the truncated gene was *wcaG* gene. In *Escherichia coli*, the gene is also referred to as *fcl* and it encodes GDP fucose synthetase [33]. The homologue of *wcaG* is at PC1_01313 from *P. carotovorum* PC1 and PC21_013440 from *P. carotovorum* PC21 genomes. In Ecc71 *wcaG* orf is 960 base pairs (bp) long and encodes 320 aa protein. It is 96% and 88% identical to the *wcaG* gene of *P. carotovorum* strains PCC21 and PC1, respectively. Gene *wcaG_Ecc71_* has 99%, 99%, 78% sequences identity and its predicted product WgaG_Ecc71_ had 100%, 100%, 99% identity with their respective homologues from *P. carotovorum* PC1, *P. carotovorum* PCC21 and *Escherichia coli K-12* sub-strain *MG1655*, respectively (Figure 5B). The multiple alignments of the deduced amino acid sequence of *wcaG* from *P. carotovorum* Ecc71 was carried out with those from *P. carotovorum* PC1, *P. carotovorum* PCC21, and *Escherichia coli K-12* (sub-strain *MG1655*). Both the multiple sequence alignment and phylogenetic tree (Figure 5A,B) show a close relationship among *P. carotovorum* PC1, *P. carotovorum* PCC21, and *Escherichia coli K-12*.

### 3.3. Pathogenicity Assays and Bacterial Population in Planta

**Figure 6:**
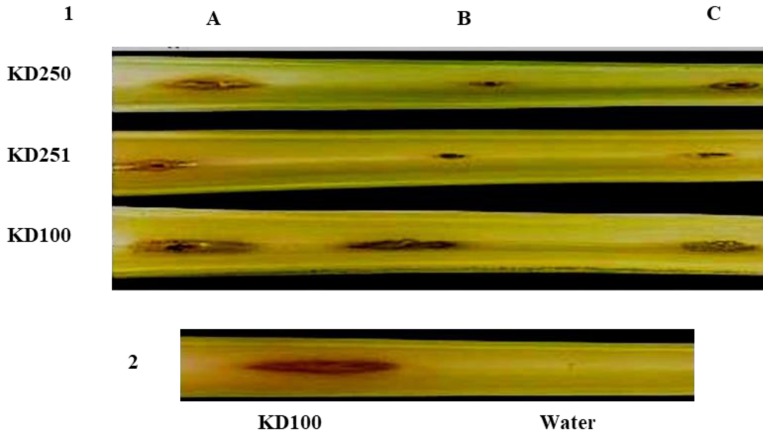
Celery petioles maceration by *P. carotovorum* strain KD100 and its *wcaG* mutants KD250 and KD251. Infection of KD250, KD251 was compared with parent KD100 and also with complemented mutants. (1) Column (**A**) indicates infection of KD250, KD251, and KD100 carrying extrachromosomal copies of *wcaG* in pCKD252; Column (**B**) infections of KD250, KD251, and KD100 carrying pLAFR5; Column (**C**) infections of KD250, KD251, and KD100. (2) Maceration caused by strain KD100 was compared with the negative control.

**Figure 7:**
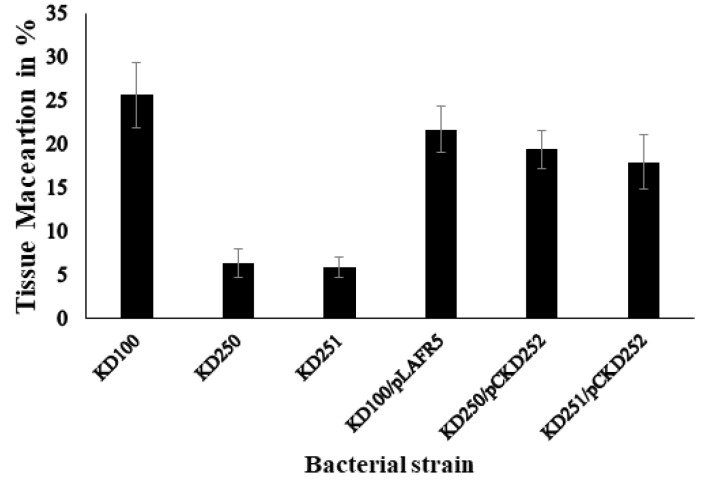
Tissues maceration capacity of each mutant was compared to the parental strain KD100. Bacteria cells were inoculated with 1.3 × 10^9^ CFU/mL in carrot disks and weight of macerated tissue was determined after 48 h of incubation at 28 °C. Data represents mean percentage weights of macerated tissue (from four replicates) ± standard deviation.

**Figure 8:**
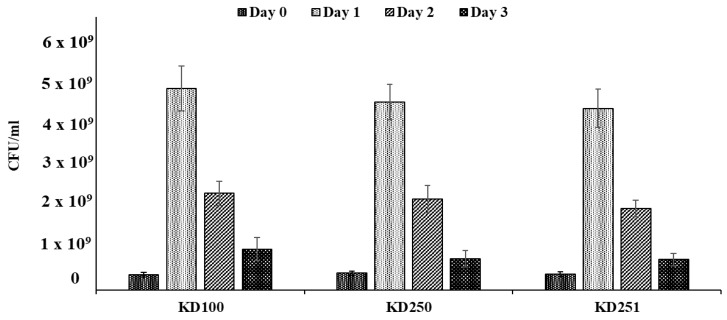
Survival capacity of *P. carotovorum* strains. Bacterial population was measured in celery petioles to determine the survival response in the host plant. Approximately 3.8 × 10^8^ CFU/mL of bacterial suspension was used to inoculate celery petioles. Bacterial cell count was taken of infected celery on days 0, 1, 2, and 3 post inoculation. Values are the mean (from three replicates) ± standard deviation.

The insertion of the transposon in two locations and therefore disruption *wcaG* gene in *P. carotovorum* led to reduction in the production of exoenzymes. Therefore, we wanted to determine if the levels of exoenzymes would affect tissue maceration as well. Both *wcaG* mutants macerated less tissue of celery petioles and carrot root discs in comparison with the parental strain. Figure 6 shows that, 24 h. post inoculation, the mutants visibly macerated less celery petioles relative to their parental strain. We measured the amount of macerated tissue of carrot root disc to quantify the differences seen in virulence between *wcaG^−^* mutants and their parent. The amounts of macerated tissue in carrot discs inoculated with parent strain KD100 were 21.5% and 26.7%, respectively, of the original weight of the disc. By contrast, their respective *wcaG^−^* mutants KD250 and KD251, macerated only 6.4% and 5.8%, respectively (Figure 7). We also considered whether besides the effects on exoenzymes production, *wcaG* mutants might also be negatively affected in multiplication or survival in the host plant tissues. For this, we checked survival and multiplication ability of *wcaG* mutants and their parent in celery petioles. We recovered similar levels of bacteria in macerated tissues of the *wcaG^−^* mutant to the parent after starting with approximately the same levels of inocula (Figure 8). This indicates that the mutation did not affect the survival and multiplication ability of the pathogen.

### 3.4. Measurement of the Cell Surface Properties

**Figure 9:**
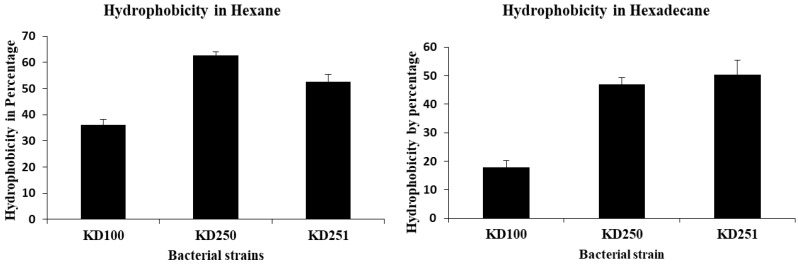
Cell surface hydrophobicity of *P. carotovorum* strains. Hydrophobicity was compared between mutants and parent. Bacterial suspensions in 0.5× PBS (aqueous phase) were mixed with non-polar solvent hexane and hexadecane. The bars represent the percentages of bacteria remaining in the non-polar phase in contrast to aqueous phase. Values are the mean (from three replicates) ± standard deviation.

**Figure 10:**
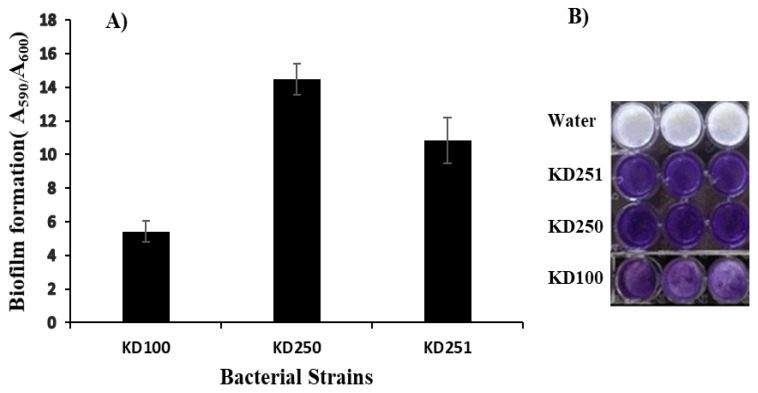
(**A**) Quantitative detection of biofilm formation in polystyrene 96-well plates. Biofilm formation was determined using the absorbance ratio of A_590_ and A_600_. (**B**) Visualization of biofilm formation by crystal violet staining. Biofilm in microtiter wells were observed with crystal violet staining after incubation of bacterial strains for 20 h at 28 °C (More than three microtiter wells were used to quantify biofilm production for each bacterial strain, Values are the mean ± standard deviation).

Previous studies in *Escherichia coli* had shown that defects in colanic acid biosynthetic genes alter the cell surface properties including hydrophobicity and adhesion [34,35]. We therefore considered whether WcaG^−^ mutants of *Pectobacterium* might also be similarly affected in cell surface properties such as hydrophobicity and biofilm formation in vitro. We measured cell hydrophobicity in vitro using two non-polar solvents, hexane and hexadecane in contrast to water. Both *wcaG* mutants showed more hydrophobicity relative to their parent (Figure 9). WcaG deficiency caused about two-fold higher hydrophobicity compared to their parental *wgaG^+^* strains. In *Escherichia coli* it has been shown that colanic acid is necessary for the development of typical biofilm activity [36]. We wanted to check whether the defect in the *wcaG* gene had any effect in biofilm formation of mutants. We measured biofilm activities using the crystal violet method as previously described [30]. Surprisingly, both *wcaG* mutants produce more biofilm than their parent KD100 (Figure 10).

## 4. Discussion

In this study, we showed through several lines of evidence that the gene, *wcaG*, is involved in virulence and cell surface properties in *Pectobacterium carotovorum*. *wcaG* encodes NAD-dependent epimerase/dehydratase, an enzyme involved in colanic acid biosynthesis. First, two transposon mutants with insertions in different regions of the gene had a similar phenotype. They both produce low levels of PCWDE, which act as the main virulence factors of the organism. Second, consistent with the role of PCWDEs in virulence, the mutants had reduced virulence in comparison with their parent. Third, the mutants were similarly affected in the cell surface properties of biofilm formation and hydrophobicity. Finally, extra-chromosomal copies of the genomic segment containing the wild type and functional *wcaG* gene restored the mutant phenotype back to parental levels.

The mutants produced less PCWDE than the parental strain. The levels of all the major PCWDEs, pectate lyase, polygalacturonase, cellulose, and protease were reduced in both mutants. Such mutants, which are globally affected in PCWDE production, tend to be mutants in regulatory genes that control the expression of enzyme genes. However, the product of *wcaG* has been annotated as an enzyme involed in colonic acid biosynthesis. It is not immediately clear at what level of gene expression PCWDE production is affected in the mutants. Enzyme production could be affected at the transcriptional, post-transcriptional, translational, or at the secretion level. However, the fact that different enzymes, protease, cellulose, and pectinase are affected suggests that the effect of the mutation might not be through secretion. The affected enzymes are secreted through at least two different secretion pathways. While protease is secreted through type I secretion system, pectate lyase, polygalacturonase, and cellulase are all secreted through type II secretion system [4,37,38]. We therefore think it is improbable that the *wcaG* mutation is affecting both type I and type II secretion systems. We have initiated experiments to determine among other things, whether the production of exoproteins such as harpins which are secreted through the type III pathway is equally affected in *wcaG* mutants. Further studies are also underway to measure promoter activity and the levels of transcripts or enzyme genes.

In the pathogenicity test, both mutants, KD250 and KD251 were less virulent than the parental strain, KD100 in host tissue maceration. This was expected since the mutants produced reduced levels of exoenzymes. Recently it has been shown that deletion of *wcaJ*, another gene whose product is involved in colonic acid (CA) biosynthesis in *Edwardsiella tarda*, a Gram-negative bacterium, leads to reduced virulence [39]. Colanic acid has also been shown to act as avirulence factor for many Enterobacteriaceae including, *E. amylovora*, *Salmonella* [16,40,41] and to also have a high influence in the production of slime layer, capsule, and biofilm [42,43]. Studies in *Erwinia amylovora* have demonstrated that mutants of two-component *rcs* regulatory system, *rcsB*, *rcsC*, and *rcsD* are affected in CA biosynthesis and these mutants are non-pathogenic on immature pear fruit [44]. This suggests that CA contributes to pathogenesis on host plant in *E. amylovora*. In *Escherichia coli*, CA plays a role in protecting the bacterium from unfavorable pH [45]. Consistent with this protective role, unfavorable conditions including low temperature and oxidative stress upregulate CA biosynthesis in *Escherichia coli* [41]. In this study, we did not study the survival of the mutants and parent under various adverse conditions including pH. We are therefore unable to speculate if CA might have a similar role in host infectivity in *Pectobacterium* although the parent and mutants colonized host tissues to the same levels. As a result, we cannot tell whether the observed effect of *wcaG* mutation on virulence is through the observed low enzyme production or through reduced, modified, or deficient colonic acid production.

The Rcs phosphorelay signal transduction system regulates biosynthesis of CA [46,47]. The sensor kinases, RscC and RscD perceive and transmit the environmental signal through simultaneous phosphorylation and dephosphorylation with the response regulator, RscB, in a process that also involves the ATP-dependent Lon protease. Interestingly, this same Rcs system has also been demonstrated to regulate virulence factors in *Pectobacterium* [48]. The Rcs system negatively regulates PCWDEs production indirectly via RcsB’s negative effect on FhlDC and RsmB but not directly on PCWDE genes [49]. However, while Rcs genes code for regulators, the mutant gene in KD250 and KD251 is a structural gene that encodes an enzyme, NAD-dependent epimerase/dehydratase. We therefore cannot speculate how its deficiency will result in a global negative effect on PCWDE production.

The WcaG mutants of *Pectobacterium* acquired cell surface properties of higher cell surface hydrophobicity and ability to form biofilms. Neither the wild type Ecc71 nor the parental strain, KD100 form any significant amount of biofilm on abiotic surfaces. This suggests that the mutant cell surfaces became more hydrophobic and therefore able to stick together and on other surfaces. We are not sure whether disruption of *wcaG* in this study completely blocked CA biosynthesis. However, hydrophobicity testing gave us some clues because the mutants were more hydrophobic than the parent. We speculate that, defective CA biosynthesis resulted in immature CA production which causes loss of acid function and so hydrophilicity. This gain of functions such as biofilm formation and hydrophobicity by the mutants contrasts with the loss of PCWDE production and virulence by the mutants. Attachment and cell surface hydrophobicity are often required for full virulence in organisms such as *P. aeruginosa* and *Xanthomonas axonopodis* pv. *citri* [50]. In addition, CA plays a significant role during development of biofilm on the biotic host surface of *Salmonella* [42] where attachment is important. In *Escherichia coli*, CA is essential for building a multidimensional structure of biofilm development although it does not play a role in the initial attachment stage during the biofilm production [36]. Interestingly, in *P. carotovorum* mutants described in this study, these properties are rather associated with loss of virulence. Recently, it has been shown that transposon insertion in *wcaG* and several other CA biosynthetic genes in another strain of *Pectobacterium carotovorum* led to resistance to infection by phage POP72 in the family *Podoviridae* [51]. Unfortunately, these phage-resistant mutants were not tested for PCWDE production or their surface properties, beside susceptibility to phage infection. We are therefore not able to learn much from those mutants on the effect of colonic acid on virulence in *Pectobacterium*.

In conclusion, we demonstrated in this study that WcaG plays a role in PCWDE production, cell surface properties, and virulence in *Pectobacterium carotovorum*. *Pectobacterium carotovorum* strains with mutations in *wcaG* produced less PCWDEs, had more hydrophobic cell surfaces, formed more biofilm on abiotic surfaces, and were ultimately impaired in host tissue maceration. Further confirmation of the WcaG role comes from the restoration of these phenotypes through complementation with wild type *wcaG^+^* clones. Further investigation is required to determine how a mutation in EPS biosynthetic gene would produce such global changes in *Pectobacterium*.

## Figures and Tables

**Table 1:** Bacterial Strains or Plasmids.

Bacterial Strain	Relevant Characteristics	References
*Pectobacterium carotovorum*		
Ecc71	Wild type	[21]
AC5006	*Lac^−^* mutant of Ecc71	[22]
KD100	Nal^r^ derivative of AC5006	This study
KD250	*wcaG*^−^ Km^r^ derivative of KD100 by mini-Tn5-Km *lacZ1* mutagenesis	This study
KD251	*wcaG^−^* Km^r^ derivative of KD100 by mini-Tn5-Km *lacZ1* mutagenesis	This study
*Escherichia coli*		
HB101	*proA*1 *lacY hsdS*20 (rB^−^ mB^−^) *recA56 rpsL20*	[23]
LE392	*McrA*^−^*hsdR514* supE44 supF58 *lacY1* or D(*lacIZY*)6 *galK2 galT22 metB1 trpR55*	Promega
S17-1	F2 *pro recA1* rB^−^ mB^+^ RP4-2 integrated (Tc::Mu) (Km::Tn*7*[Smr Tpr])	[24]
Plasmids		
pUT mini-Tn5*lacZ*1	A λ-Pir vector containing mini-Tn5-Km l*acZ*1 transposon	[25]
pRK2013	IncP Kmr TraRk2^+^ D*repRK*2 *repE*1	[26]
pLAFR5	Tcr, cosmid cloning vector	[27]
pCKD252	*wcaG^++^* cosmid in pLAFR5	This study

## Supplementary Materials

The following are available online at https://www.mdpi.com/2076-2607/7/6/172/s1, Table S1: Names and sequences of oligonucleotide primers used in this study.
