# Supplementary material for: The Gene Encoding NAD-Dependent Epimerase/Dehydratase, wcaG, Affects Cell Surface Properties, Virulence, and Extracellular Enzyme Production in the Soft Rot Phytopathogen, Pectobacterium carotovorum
Source: Microorganisms. 2019 Jun 13;7(6):172. doi: 10.3390/microorganisms7060172 (PMC6616942; doi:10.3390/microorganisms7060172)
Supplement: Supplementary file 1 [file microorganisms-07-00172-s001.pdf]

Table S1. Primers sequences used for *wcaG* amplification.

Primer Name	Primer sequence	Primers Pair	Size(bp) of amplified *wcaG*
A_71_NAD_F	5′CAGGACGCAGTCAAAAGTTTC3′	AD	670
B_71_NAD_F	5′AAACTTGTTGAACAGCCGATG3′	AE	490
C_71_NAD_F	5′TGATAACTTCCATCCCGAAAA3′	BD	465
D_71_NAD_R	5′TTGGTATCCAACAACCTTTGC3′	BE	290
E_71_NAD_R	5′TCGGTTTACCACTACCCCATA3′	CE	110
E_71_NAD_R	5′GGCAATCTTGGCAATAGCATA3′	CD	290
TnLacZ-P6	TCGGTTGTACAAAAACTTTC		
